# Population Pharmacokinetics and Pharmacodynamics of Chloroquine in a *Plasmodium vivax* Volunteer Infection Study

**DOI:** 10.1002/cpt.1893

**Published:** 2020-07-02

**Authors:** Azrin N. Abd‐Rahman, Louise Marquart, Nathalie Gobeau, Anne Kümmel, Julie A. Simpson, Stephan Chalon, Jörg J. Möhrle, James S. McCarthy

**Affiliations:** ^1^ QIMR Berghofer Medical Research Institute Brisbane Australia; ^2^ Medicines for Malaria Venture Geneva Switzerland; ^3^ IntiQuan GmbH Basel Switzerland; ^4^ Centre for Epidemiology and Biostatistics Melbourne School of Population and Global Health The University of Melbourne Melbourne Australia; ^5^ University of Queensland Brisbane Australia

## Abstract

Chloroquine has been used for the treatment of malaria for > 70 years; however, chloroquine pharmacokinetic (PK) and pharmacodynamic (PD) profile in *Plasmodium vivax* malaria is poorly understood. The objective of this study was to describe the PK/PD relationship of chloroquine and its major metabolite, desethylchloroquine, in a *P. vivax* volunteer infection study. We analyzed data from 24 healthy subjects who were inoculated with blood‐stage *P. vivax* malaria and administered a standard treatment course of chloroquine. The PK of chloroquine and desethylchloroquine was described by a two‐compartment model with first‐order absorption and elimination. The relationship between plasma and whole blood concentrations of chloroquine and *P. vivax* parasitemia was characterized by a PK/PD delayed response model, where the equilibration half‐lives were 32.7 hours (95% confidence interval (CI) 27.4–40.5) for plasma data and 24.1 hours (95% CI 19.0–32.7) for whole blood data. The estimated parasite multiplication rate was 17 folds per 48 hours (95% CI 14–20) and maximum parasite killing rate by chloroquine was 0.213 hour^−1^ (95% CI 0.196–0.230), translating to a parasite clearance half‐life of 4.5 hours (95% CI 4.1–5.0) and a parasite reduction ratio of 400 every 48 hours (95% CI 320–500). This is the first study that characterized the PK/PD relationship between chloroquine plasma and whole blood concentrations and *P. vivax* clearance using a semimechanistic population PK/PD modeling. This PK/PD model can be used to optimize dosing scenarios and to identify optimal dosing regimens for chloroquine where resistance to chloroquine is increasing.

**Study Highlights**

**WHAT IS THE CURRENT KNOWLEDGE ON THE TOPIC?**

☑ Chloroquine has been extensively used for > 70 years; however, the relationship between chloroquine pharmacokinetic (PK) and parasite killing has not been quantified using semimechanistic population PK/pharmacodynamic (PD) modeling.

**WHAT QUESTION DID THIS STUDY ADDRESS?**

☑ This study investigated the PK/PD relationship between chloroquine plasma and whole blood concentrations and *P. vivax* killing effect in a volunteer infection study using an induced blood‐stage malaria model.

**WHAT DOES THIS STUDY ADD TO OUR KNOWLEDGE?**

☑ This is the first study characterizing the PK/PD relationship of chloroquine plasma and whole blood concentrations on clearance of *P. vivax* parasites. This study demonstrates the importance of biological matrices in chloroquine PK monitoring and found a delayed effect of chloroquine on killing of *P. vivax* parasites.

**HOW MIGHT THIS CHANGE CLINICAL PHARMACOLOGY OR TRANSLATIONAL SCIENCE?**

☑ The developed PK/PD model provides a valuable *in silico* prediction tool for determining dosing strategies that reduce the development of resistance and ensure chloroquine therapeutic effect against *P. vivax* with decreased sensitivity to chloroquine.

Globally, 225 million cases of malaria have been reported with 7.5 million cases due to *Plasmodium vivax*.^1^ Malaria causes an estimated 405,000 deaths worldwide in which 67% involve children aged under 5 years.^1^ Chloroquine was the cornerstone of malaria treatment but the spread of drug resistance has rendered chloroquine ineffective for the treatment of *P. falciparum* malaria in almost all regions of the world. However, chloroquine remains a valuable antimicrobial agent for the treatment of *P. vivax* malaria in most countries because of its low cost and well‐understood safety profile.

Chloroquine resistance to *P. vivax* emerged in Papua New Guinea in the late 1980s,^2^, ^3^ and evidence of reduced chloroquine efficacy has been reported in other malaria endemic countries.^4^, ^5^ Evaluating the efficacy of antimalarial drugs in *P. vivax* malaria is difficult due to lack of a continuous *in vitro P. vivax* culture system, and by the inability of molecular genotyping to discriminate between recrudescence (i.e., blood‐stage treatment failures), relapse, and new infections.

Inadequate chloroquine dosing leads to subtherapeutic exposure and increased risk of treatment failure and resistance development. The pharmacokinetic (PK) and pharmacodynamic (PD) properties of chloroquine and its active metabolite, desethylchloroquine, in *P. vivax* malaria have been characterized in only a few studies.^6^, ^7^, ^8^, ^9^, ^10^, ^11^, ^12^, ^13^, ^14^ Of note, the relationship between the PK profile of chloroquine and parasite killing has not been described using semimechanistic PK/PD modeling approaches. Knowledge on the PK profile of chloroquine and its metabolite, as well as their antimalarial effect against *P. vivax*, would provide a valuable tool for clinical prediction of the efficacy of various dosing regimens, and for optimizing the current dosing regimen, particularly to combat rising resistance to chloroquine.

In this study, we investigated the PK/PD relationship of chloroquine and desethylchloroquine by analyzing data from a volunteer infection study (VIS) using the *P. vivax* induced blood‐stage malaria model.^15^ We used nonlinear mixed effects modeling to characterize the PK and PD properties of chloroquine and desethylchloroquine. The results from our modeling will help optimizing chloroquine dosing regimens in *P. vivax* malaria field studies.

## METHODS

### Study design and subjects

Study design, procedure, and main results were described in detail elsewhere.^15^ Briefly, the study was a phase Ib, single‐center, open‐label trial (ACTRN12616000174482) in three cohorts of eight subjects each. The 24 healthy subjects were inoculated with ~ 564 viable *P. vivax*‐infected erythrocytes administered intravenously on day 0. Chloroquine was administered as chloroquine phosphate tablet (Avloclor). Subjects received a total chloroquine dose of 1.55 g base for ≥ 60 kg adults or 25 mg base/kg for < 60 kg adults orally over 3 days on day 8, 9, or 10. The study was approved by the QIMR Berghofer Medical Research Institute Human Research Ethics Committee and the Australian Defence Human Research Ethics Committee. All subjects provided written informed consent before participating.

Samples for measuring chloroquine and desethylchloroquine concentrations in plasma and whole blood were collected before chloroquine administration and at 1, 2, 3, 4, 6, 12, 24, 48, 72, 96, 168, 240, and 480 hours after first dose administration. Blood samples for *P. vivax* counts were collected before inoculation, daily from day 4 until parasitemia was detected, twice‐daily thereafter until treatment day, before first dose on treatment day, and at 2, 4, 8, 12, 16, 24, 30, 36, 48, 60, 72, 96, 120, 168, 240, and 480 hours after the first dose.

Chloroquine and desethylchloroquine concentrations were measured using liquid chromatography tandem mass spectrometry. The lower limit of quantification (LLOQ) was 1.35 µg/L for chloroquine plasma, 1.0 µg/L for whole blood chloroquine, 1.3 µg/L for plasma desethylchloroquine, and 1.75 µg/L for whole blood desethylchloroquine concentrations. *P. vivax* parasitemia was measured using quantitative real‐time polymerase chain reaction targeting the 18S rRNA gene.^16^ The limit of detection (LOD) was 10 parasites/mL.

### Data used for analysis

The population PK/PD analyses included 1,084 drug concentration measurements (271 for each chloroquine and desethylchloroquine in plasma and whole blood) and 619 *P. vivax* parasitemia measurements. Of these measurements, 8.9% of chloroquine plasma, 11.8% of desethylchloroquine plasma, 8.9% of chloroquine whole blood, and 10% of desethylchloroquine whole blood concentrations were below the LLOQ. Chloroquine and desethylchloroquine concentrations in erythrocytes were calculated and correlations between drug concentrations in whole blood and erythrocytes were determined (see **Supplementary Material** **S1**). Approximately 25.5% of parasitemia levels were below the LOD, of which 26% were from measurements taken before chloroquine treatment. The remainder of parasitemia levels below the LOD (74%) occurred at median of 66 hours (range 24–120) after the first dose of chloroquine. Recrudescence was not observed in any subject over the study period.

### Pharmacometric modeling

Nonlinear mixed effects modeling was performed using Monolix version 4.3.3 (Lixoft, Antony, France). For data programming, preprocessing and postprocessing of the modeling, and simulations MATLAB R2015b (MathWorks, Natick, MA) and the MATLAB toolbox IQMtools version 1.2.2.2 (IntiQuan GmbH, Basel, Switzerland) was used. The graphic processing of the Monolix output for the final model was performed with R (version 3.5.1)^17^ and ggplot2 (version 3.0.0).^18^

#### Pharmacokinetic modeling

Chloroquine and desethylchloroquine plasma and whole blood concentrations were converted into molar units using the molecular masses of 319.8 and 291.8 g/mol, respectively. The chloroquine phosphate doses were converted to the equivalent (base) chloroquine dose by multiplying chloroquine phosphate by 0.62 and converted into molar units. Correlations between chloroquine (Spearman’s rho = 0.99) and desethylchloroquine (Spearman’s rho = 0.98) whole blood and erythrocyte concentrations were high (**Figure** **S1**). Given the strong relationship between whole blood and erythrocyte concentrations, whole blood concentration was used as a surrogate for the parasite killing effect. PK modeling was conducted separately for plasma and whole blood concentration‐time data. First, a population PK model of chloroquine was developed and then the PK model was extended to describe concurrent PK of chloroquine and desethylchloroquine. Desethylchloroquine is known to have a killing activity against chloroquine‐sensitive *P. falciparum* strains.^19^, ^20^, ^21^, ^22^ Based on the assumption that desethylchloroquine antimalarial activity is similar against *P. vivax*, desethylchloroquine PK data were incorporated into the PK model with the aim to be used in the PK/PD model. Data below the LLOQ were treated using the left‐censoring method, by maximizing the likelihood of the data being below LLOQ.^23^

One‐compartment, two‐compartment, and three‐compartment PK models with different absorption (zero‐order, first‐order, and simultaneous and sequential mixed zero‐order and first‐order with or without lag time) and elimination (linear and parallel linear and saturable) models were tested. Interindividual variability (IIV) was assumed to be log‐normally distributed. Correlations between PK parameters were evaluated by estimating the off‐diagonal components of the variance‐covariance matrix. Interoccasion variability related to $F_{\text{rel}}$ was tested for the second, third, and fourth doses vs. the first dose. Additive, proportional, and combined error models were tested to explain random unexplained variability (see **Supplementary Material** **S1**).

Age, sex, and body weight were tested during covariate model building. Effect of covariates on PK parameters was evaluated with a stepwise forward‐addition (*P* < 0.05) and backward‐deletion (*P* < 0.01) approach. Continuous covariates were modeled as power function and categorical covariates as linear function (see **Supplementary Material** **S1**). Covariates were selected based on biological plausibility, statistical significance, and clinical relevance. A covariate was retained in the final model if its contribution was clinically important, which was defined as 95% confidence interval (CI) of the covariate effect lying completely outside of a predetermined limit (±20%).

#### Pharmacokinetic‐pharmacodynamic modeling

Each *P. vivax* parasitemia measurement was quantified in replicate per time points and log_10_‐transformed. The mean of the log_10_‐transformed parasitemia per time point per subject was used in the analysis. A sequential PK/PD modeling approach was used in which the empirical Bayes estimates of individual PK parameters from plasma and whole blood samples were used as regression parameters. The initial aim was to include the antimalarial effect of desethylchloroquine in the PK/PD model; however, desethylchloroquine was excluded from the analysis as it was assumed that its contribution to the parasite killing effect was minor based on its low concentrations in both plasma and whole blood. The low exposures of desethylchloroquine relative to chloroquine suggest that chloroquine contributes most to the antimalarial effect. The left censoring method was used for parasitemia below the LOD.

As the maximum parasite killing rate of chloroquine ( $E_{\max\text{CQ}}$) is independent of whether plasma or whole blood concentrations were measured in describing the parasite killing effect, a common $E_{\max\text{CQ}}$ parameter was used to fit the two models simultaneously. To technically enable the simultaneous fit, the observed parasitemia levels were duplicated to link each set separately to the empirical Bayes estimates of the PK parameters for plasma or whole blood concentrations. We tested several PD models, including a direct effect ( $E_{\max}$), a turnover, and an effect compartment model (see **Supplementary Material** **S1**). The IIV of each PD parameter was described using a log‐normal variance model, except for the log_10_‐transformed baseline parasitemia, for which a normal distribution was assumed. An additive error model was used to present the residual random unexplained variability in the log_10_ parasitemia measurements. Secondary parameters, such as parasite multiplication rate over 48 hours ( ${\text{PMR}}_{48}$), parasite reduction ratio over 48 hours ( ${\text{PRR}}_{48}$), parasite clearance half‐life ( ${\text{PCt}}_{1/2}$), and time above chloroquine plasma and whole blood concentration at half of maximum effect ( ${\text{EC}}_{50\;\text{CQ biological matrix}}$) were estimated using individual PD parameters obtained via empirical Bayes estimates (see **Supplementary Material** **S1**).

#### Model evaluation

The appropriateness of the model was evaluated using basic goodness‐of‐fit diagnostics and visual predictive check (VPC). A VPC was performed by simulating 1,000 observations at each time point and 95% CI of the 5th, 50th, and 95th simulated percentiles were plotted against observations. In addition, successful convergence of the minimization routine, reasonable physiological plausibility, and precision of parameter estimates were also taken into account during the model development.

#### Simulations of chloroquine resistance

Simulations were performed to evaluate different mechanisms of chloroquine resistance on treatment success at day 28 by varying $E_{\max\text{CQ}}$, ${\text{EC}}_{50\text{CQ plasma}}$, and ${\text{EC}}_{50\;\text{CQ whole blood}}$ values (see **Supplementary Material** **S1**).

## RESULTS

All subjects from the previously reported clinical trial (three cohorts, *n* = 8 per cohort) were included in the analysis.^15^ **Table** **1** shows the demographic characteristics of the subjects, chloroquine dose, and treatment day. The majority of the subjects (91.7%) received 1,000 mg chloroquine phosphate initially, followed by 500 mg or 375 mg at 6, 24, and 48 hours after the first dose administration. Chloroquine treatment commenced on day 8 for cohort 1 and on day 10 for cohorts 2 and 3, except for one subject (subject 205) who commenced treatment on day 9. Plasma, whole blood, and erythrocyte concentrations of chloroquine and desethylchloroquine time profiles are presented in **Figure** **S1**.

**Table 1:** Demographic characteristics, treatment day, and chloroquine dose for all subjects

Characteristics	Subjects analyzed (*n* = 24)
Sex, *n* (%)	
Male	13 (54.2)
Female	11 (45.8)
Age, years	
Mean (SD)	25.6 (6.6)
Range	19–44
Race, *n* (%)	
White	21 (87.5)
Indigenous aboriginal	1 (4.2)
Latino	1 (4.2)
Asian	1 (4.2)
Weight, kg	
Mean (SD)	73.3 (10.8)
Range	57.2–99.5
Height, cm	
Mean (SD)	175.8 (9.8)
Range	156–190
Body mass index, kg/m^2^	
Mean (SD)	23.7 (2.7)
Range	19.1–29.5
Treatment day, *n* (%)	
Day 8	8 (33.3)
Day 9	1 (4.2)
Day 10	15 (62.5)
CQ base doses^a^	
0 hours	620 mg (*n* = 24)
6 hours	310 mg (*n* = 24)
24 hours	310 mg (*n* = 23), 232.5 mg (*n* = 1)
48 hours	310 mg (*n* = 22), 232.5 mg (*n* = 2)
Hematocrit	
Mean (SD)	0.4 (0.03)
Range	0.31–0.51

CQ, chloroquine.

^a^ Chloroquine was administered as chloroquine phosphate (Avloclor). The chloroquine phosphate doses were converted to chloroquine base dose by multiplying with 0.62.

### Population pharmacokinetic modeling

**Figure 1:**
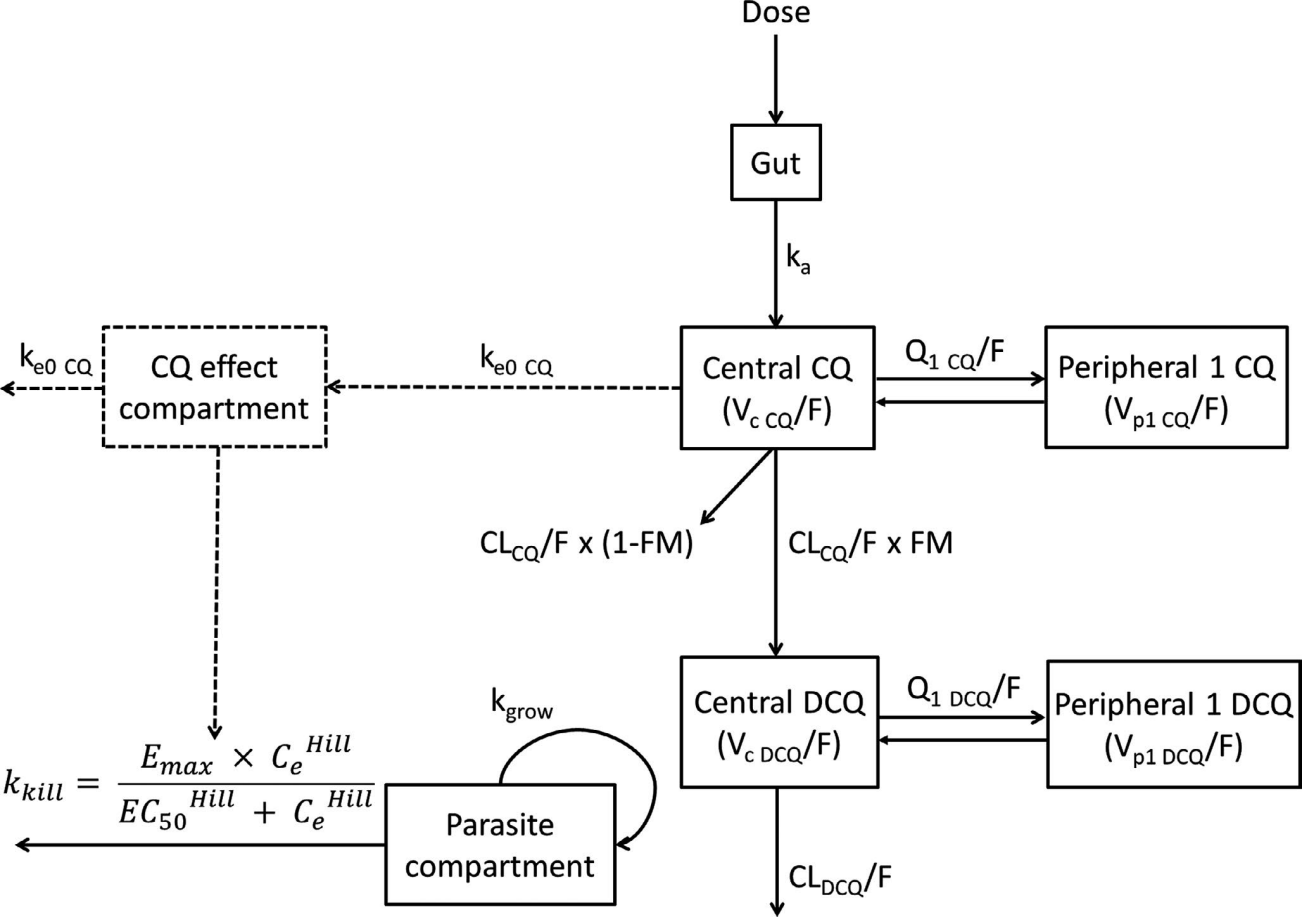
Final structural population pharmacokinetic‐pharmacodynamic model of chloroquine and desethylchloroquine in healthy subjects inoculated with blood‐stage *Plasmodium vivax*. $C_{e}$, the concentration of the drug in the hypothetical effect compartment; ${\text{CL}}_{\text{CQ}}/F$, apparent clearance of chloroquine; ${\text{CL}}_{\text{DCQ}}/F$, apparent clearance of desethylchloroquine; CQ, chloroquine; DCQ, desethylchloroquine; ${\text{EC}}_{50}$, the drug concentration producing 50% of the $E_{\max}$; $E_{\max}$, maximum parasite killing attributable to the drug; FM, fraction of chloroquine metabolized to desethylchloroquine; $F_{\text{rel}}$, relative bioavailability of first‐order absorption; Hill, the Hill coefficient; $k_{a}$, rate constant of absorption; $k_{\text{e0 CQ}}$, transfer rate constant from effect compartment of chloroquine; $k_{\text{grow}}$, growth rate of *P. vivax*; $k_{\text{kill}}$, killing rate of *P. vivax* by the drug; $Q_{1\text{CQ}}F$, apparent inter‐compartmental clearance between central compartment of chloroquine and peripheral compartment 1 of chloroquine; $Q_{1\;\text{DCQ}}F$, apparent intercompartmental clearance between central compartment of desethylchloroquine and peripheral compartment one of desethylchloroquine; $V_{\text{c CQ}}/F$, apparent volume of distribution for central compartment of chloroquine; $V_{\text{c DCQ}}/F$, apparent volume of distribution for central compartment of desethylchloroquine; $V_{\text{p1 CQ}}/F$, apparent volume of distribution for peripheral compartment one of chloroquine; $V_{\text{p1 DCQ}}/F$, apparent volume of distribution for peripheral compartment one of desethylchloroquine.

Chloroquine plasma and whole blood PK profiles were characterized by a two‐compartment model with first‐order absorption and elimination and a proportional residual error. Desethylchloroquine plasma and whole blood PK profiles were described by a two‐compartment model, with a first‐order input from the chloroquine central compartment and first‐order elimination (**Figure** **1**). Chloroquine whole blood concentrations were ~ 5.9‐fold (range 0.3–58.8) higher than plasma concentrations, whereas desethylchloroquine whole blood concentrations were ~ 7.3‐fold (range 1.5–45) higher than plasma concentrations. These results suggest a major effect of the sampling matrix analyzed. The fraction of chloroquine metabolized to desethylchloroquine (FM) and the fraction of chloroquine eliminated by other pathways (1‐FM) were not known. To allow model identifiability in this drug‐metabolite PK model, it was assumed that 18% of chloroquine is converted to desethylchloroquine, based on published estimates of the desethylchloroquine fraction of the total chloroquine dose recovered in urine.^9^, ^12^

**Table** **2** summarizes PK parameter estimates and their associated IIV. The differences observed in the parameter estimates for the volumes of distribution and clearances for plasma and whole blood samples are explained by the 6‐fold to 7‐fold average difference in concentrations derived from these sampling matrices. The elimination rate constants of chloroquine from the central compartment were similar. No covariate was included in the final PK model (see **Supplementary Material** **S1**).

**Table 2:** Chloroquine and desethylchloroquine parameter estimates from the final population pharmacokinetic models

Parameters	Plasma samples				Whole blood samples			
	Estimate	RSE (%)	95% CI	Shrinkage (%)	Estimate	RSE (%)	95% CI	Shrinkage (%)
Pharmacokinetic parameters								
$k_{a}$, hour^−1^	0.943	21	0.551–1.335	—	0.574	18	0.378–0.770	—
$F_{\text{rel}}$ (%)	100 (fixed)	—	—	—	100 (fixed)	—	—	—
${\text{CL}}_{\text{CQ}}/F$, L/h	54.6	5	49.7–59.6	—	8.96	5	8.10–9.82	—
$V_{\text{c CQ}}/F$, L	2,930	7	2,558–3,302	—	560	9	456–664	—
$Q_{1\;\text{CQ}}F$, L/h	47.2	9	38.8–55.6	—	38.5	11	30.1–46.9	—
$V_{\text{p1 CQ}}/F$, L	4,700	7	4,053–5,347	—	1,230	6	1,079–1,381	—
${\text{CL}}_{\text{DCQ}}/F$, L/h	37.6	6	32.9–42.3	—	4.42	6	3.87–4.97	—
$V_{\text{c DCQ}}/F$, L	40.0	39	8.6–71.4	—	16.1	14	11.6–20.6	—
$Q_{1\;\text{DCQ}}/F$, L/h	36.3	11	28.7–43.9	—	4.46	12	3.40–5.52	—
$V_{\text{p1 DCQ}}/F$, L	2,840	13	2,134–3,546	—	259	12	196–322	—
Interindividual variability								
$\omega_{k_{a}}$	0.662	24	0.348–0.976	18	0.467	26	0.232–0.702	22
$\omega_{F_{\text{rel}}}$	0.192	18	0.125–0.259	6	0.215	17	0.144–0.286	5
$\omega_{{\text{CL}}_{\text{CQ}}}F$	0.084	36	0.025–0.143	28	0.117	24	0.062–0.172	23
$\omega_{Vc_{\text{CQ}}}F$	0.179	27	0.085–0.273	25	0.228	29	0.097–0.359	18
$\omega_{{\text{CL}}_{\text{DCQ}}}F$	0.197	25	0.101–0.293	21	0.219	21	0.129–0.309	13
$\omega_{Vc_{\text{DCQ}}}F$	—	—	—	—	0.384	32	0.149–0.619	32
Random unexplained variability								
$\varepsilon_{\text{prop CQ}}$	0.243	5	0.219–0.267	—	0.221	5	0.199–0.243	—
$\varepsilon_{\text{prop DCQ}}$	0.347	5	0.314–0.380	—	0.253	5	0.228–0.278	—
Secondary pharmacokinetic parameters^a^, ^b^								
CQ $C_{\max}$, µmol/L	0.72 (0.25–1.38)				2.83 (1.29–4.49)			
CQ $t_{\max}$, hour	4.0 (2.0–4.8)				4.0 (2.0–5.5)			
CQ ${\text{AUC}}_{0-\inf}$, µmol∙h/L	80 (56–126)				509 (326–856)			
CQ $t_{12}$, hour	149 (136–163)				156 (124–212)			
DCQ $C_{\max}$, µmol/L	0.08 (0.03–0.22)				0.39 (0.16–0.82)			
DCQ $t_{\max}$, hour	3.3 (2.0–5.3)				4.0 (2.0–5.7)			
DCQ ${\text{AUC}}_{0-\inf}$, µmol∙h/L	21 (13–39)				233 (96–683)			
DCQ $t_{12}$, hour	104 (96–137)				83 (71–99)			

${\text{AUC}}_{0-\inf}$, area under the concentration‐time curve from zero to infinity; CI, confidence interval; ${\text{CL}}_{\text{CQ}}F$, apparent clearance of chloroquine; ${\text{CL}}_{\text{DCQ}}F$, apparent clearance of desethylchloroquine; $C_{\max}$, maximum concentration; CQ, chloroquine; DCQ, desethylchloroquine; $F_{\text{rel}}$, relative bioavailability of first‐order absorption; $k_{a}$, rate constant of absorption; $Q_{1\;\text{CQ}}F$, apparent inter‐compartmental clearance between central compartment of chloroquine and peripheral compartment 1 of chloroquine; $Q_{1\;\text{DCQ}}/F$, apparent inter‐compartmental clearance between central compartment of desethylchloroquine and peripheral compartment 1 of desethylchloroquine; RSE, relative standard error; $t_{12}$, elimination half‐life; $t_{\max}$, time to reach maximum concentration; $V_{\text{c CQ}}/F$, apparent volume of distribution for central compartment of chloroquine; $V_{\text{c DCQ}}/F$, apparent volume of distribution for central compartment of desethylchloroquine; $V_{\text{p1 CQ}}/F$, apparent volume of distribution for peripheral compartment 1 of chloroquine; $V_{\text{p1 DCQ}}/F$, apparent volume of distribution for peripheral compartment 1 of desethylchloroquine.

^a^ Secondary pharmacokinetic parameters were calculated from the empirical Bayesian *post hoc* estimates.

^b^ All values are given as median (range) unless stated otherwise.

**Figure 2:**
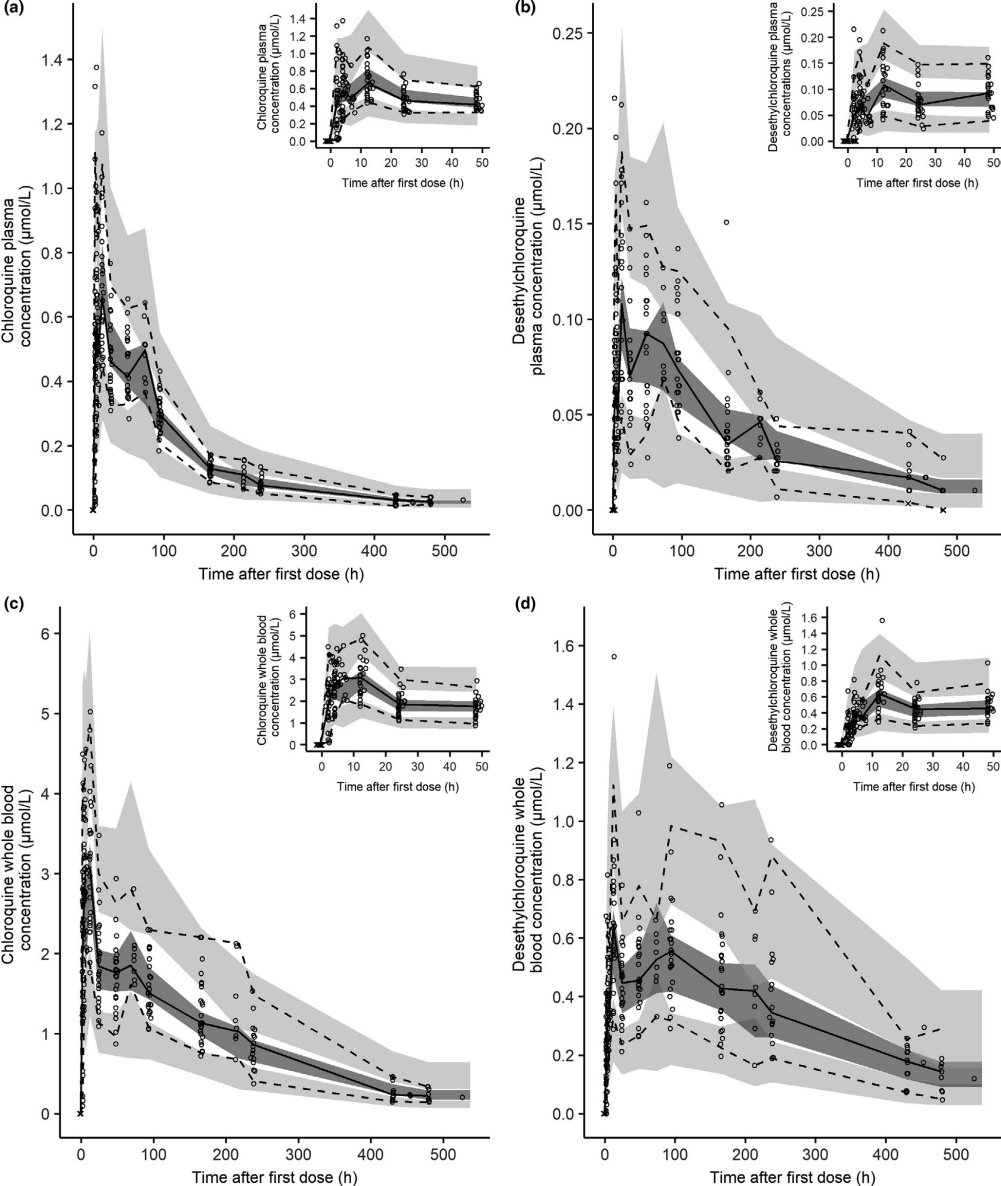
Visual predictive check of the final pharmacokinetic model of (**a**) chloroquine plasma, (**b**) desethylchloroquine plasma, (**c**) chloroquine whole blood, and (**d**) desethylchloroquine whole blood concentrations. The insets show chloroquine and desethylchloroquine simulations at 0–48 hours. The circles represent the observed data, the crosses represent the simulated values below the lower limit of quantification concentrations, the solid lines represent the 50th percentile of the observed data, the dashed lines represent the 50th and 95th percentiles for the observed data, the dark grey shaded areas represent the 95% confidence intervals for 50th percentile and the light grey shaded area the 95% confidence interval for the 5th and 95th percentiles derived from 1,000 stochastic profiles simulated from the final population pharmacokinetic model.

When the observed concentrations were compared with population and individual predicted concentrations, there was a small underprediction of the higher chloroquine and desethylchloroquine concentrations in both plasma and whole blood samples (**Figures** **S2** **–S5**). The distributions of population and individual weighted residuals (PWRES and IWRES, respectively) and normalized prediction distribution errors (NPDEs) over time showed bias in first 100 hours of drug administration (**Figures** **S2** **and** **S3**), possibly due to difficulty in modeling the absorption process using plasma data. The scatter plots of the PWRES, IWRES, and NPDE vs. population predictions (**Figures** **S2** **and** **S3**) showed positive weighted residual in the lowest population predictions, indicating a slight overprediction of the lower chloroquine and desethylchloroquine plasma concentrations. VPCs indicate that the PK models are able to reproduce the observed data (**Figure** **2**).

### Population pharmacokinetic/pharmacodynamic modeling

Individual empirical Bayesian estimates of PK parameters from plasma and whole blood data were used simultaneously to describe the drug concentration‐effect relationship. Parasite killing was described by a delayed effect of chloroquine concentration on the parasite. The equilibration half‐life for chloroquine in plasma was 32.7 hours (95% CI 27.4–40.5) and in whole blood 24.1 hours (95% CI 19.0–32.7), indicating that chloroquine concentration in the effect compartment takes some time to reach equilibrium with levels measured in plasma and whole blood. The PD parameters and their IIVs were estimated with a reasonable precision (**Table** **3**). The $E_{\max\text{CQ}}$ was estimated to be 0.213 hour^−1^ (95% CI 0.196–0.230).

**Table 3:** Chloroquine parameter estimates from the final population pharmacodynamic model

Parameter	Estimate	RSE (%)	95% CI	Shrinkage (%)
Pharmacodynamic parameters				
${\text{PL}}_{\text{base}}$, log_10_ parasite/mL	‒3.36	9	‒3.97 to (‒2.75)	—
$k_{\text{grow}}$, hour^−1^	0.059	3	0.056–0.062	—
$E_{\max\text{CQ}}$, hour^−1^	0.213	4	0.196–0.230	—
${\text{EC}}_{50\text{CQ plasma}}$, µmol/L	0.047 (fixed)	—	—	—
${\text{EC}}_{50\text{CQ whole blood}}$, µmol/L	0.28 (fixed)	—	—	—
${\text{Hill}}_{\text{CQ plasma}}$	2.5 (fixed)	—	—	—
${\text{Hill}}_{\text{CQ whole blood}}$	2.5 (fixed)	—	—	—
$k_{e0\text{CQ plasma}}$, hour^−1^	0.0212	10	0.0171–0.0253	—
$k_{e0\;\text{CQ whole blood}}$, hour^−1^	0.0288	14	0.0212–0.0364	—
Interindividual variability				
$\omega_{{\text{PL}}_{\text{base}}}$	1.47	22	0.843–2.097	11
$\omega_{k_{\text{grow}}}$	0.137	18	0.090–0.184	13
$\omega_{E_{\max\text{CQ}}}$	0.193	7	0.168–0.218	4
$\omega_{{\text{EC}}_{50\text{CQ plasma}}}$	0.3 (fixed)	—	—	—
$\omega_{{\text{EC}}_{50\text{CQ whole blood}}}$	0.3 (fixed)	—	—	—
$\omega_{k_{e\text{CQ plasma}}}$	0.1 (fixed)	—	—	—
$\omega_{k_{e\;\text{CQ whole blood}}}$	0.1 (fixed)	—	—	—
Random unexplained variability				
$\varepsilon_{\text{add plasma}}$	0.378	2	0.365–0.391	—
$\varepsilon_{\text{add whole blood}}$	0.379	3	0.357–0.401	—
Secondary pharmacodynamics parameters^a^				
	**Geometric mean (95% CI)**		**Median (range)**	
${\text{PMR}}_{48}$, parasite/48 h	17.4 (14.3–20.4)		16.1 (10.3–41.0)	
${\text{PRR}}_{48\;\text{plasma}}$, log_10_ difference	2.6 (2.5–2.7)^b^		2.7 (2.3–2.9)	
${\text{PRR}}_{48\;\text{whole blood}}$, log_10_ difference	2.6 (2.5–2.7)^b^		2.6 (2.2–2.8)	
${\text{PCt}}_{1/2}$, hour	4.5 (4.1–5.0)		4.7 (2.2–6.7)	
Time above ${\text{EC}}_{50\text{CQ plasma}}$, days	14.3 (13.5–15.1)		14.2 (10.7–17.3)	
Time above ${\text{EC}}_{50\text{CQ whole blood}}$, days	18.3 (17.0–19.6)		18.1 (14.4–26.3)	

CI, confidence interval; CQ, chloroquine; ${\text{EC}}_{50\text{CQ biological matrix}}$, chloroquine concentration in biological matrix that produces 50% of the E_max_; $E_{\max\text{CQ}}$, maximum parasite killing rate by chloroquine; ${\text{Hill}}_{\text{CQ biological matrix}}$, Hill coefficient reflecting the steepness of the chloroquine concentration in biological matrix‐effect relationship; $k_{e0\;\text{CQ biological matrix}}$, transfer rate constant to and from effect compartment of chloroquine in biological matrix; $k_{\text{grow}}$, parasite growth rate; ${\text{PCt}}_{1/2}$, parasite clearance half‐life; ${\text{PL}}_{\text{base}}$, parasitemia level at baseline; ${\text{PMR}}_{48}$, parasite multiplication rate per 48 hours; ${\text{PRR}}_{48\;\text{biological matrix}}$, parasite reduction ratio in biological matrix per 48 hours; RSE, relative standard error.

^a^ Secondary pharmacodynamic parameters were calculated from the empirical Bayesian *post hoc* estimates.

^b^ Values are arithmetic mean (95% CI).

Sensitivity analysis revealed that the estimates of some parameters changed substantially with different initial condition values: ${\text{EC}}_{50\;\text{CQ plasma}}$, ${\text{EC}}_{50\;\text{CQ whole blood}}$; transfer rate constant of chloroquine plasma, and whole blood concentration from and to the central compartment and an effect compartment ( $k_{e0\;\text{CQ plasma}}$, $k_{e0\;\text{CQ whole blood}}$). These parameters were difficult to estimate because none of the subjects had recrudescence. As a result, we fixed ${\text{EC}}_{50\;\text{CQ whole blood}}$ to 0.28 μmol/L, a value previously reported in the literature based on relapse data.^14^ We assumed that the ${\text{EC}}_{50\;\text{CQ plasma}}$ is six folds lower than ${\text{EC}}_{50\;\text{CQ whole blood}}$, based on the whole blood‐to‐plasma ratio of chloroquine concentrations in this study. The geometric mean of estimated ${\text{PMR}}_{48}$ was 17.4‐fold per 48 hours (95% C: 14.3–20.4). The arithmetic mean of ${\text{PRR}}_{48}$ was the same between plasma and whole blood (2.6; 95% CI 2.5–2.7), corresponding to a PRR of 400 every 48 hours (95% CI 320–500). The geometric mean of estimated ${\text{PCt}}_{1/2}$ was 4.5 hours (95% CI 4.1–5.0). The geometric mean of estimated time above ${\text{EC}}_{50\;\text{CQ plasma}}$ was 14.3 days (95% CI 13.5–15.1) and time above ${\text{EC}}_{50\;\text{CQ whole blood}}$ was 18.3 days (95% CI 17.0–19.6).

**Figure 3:**
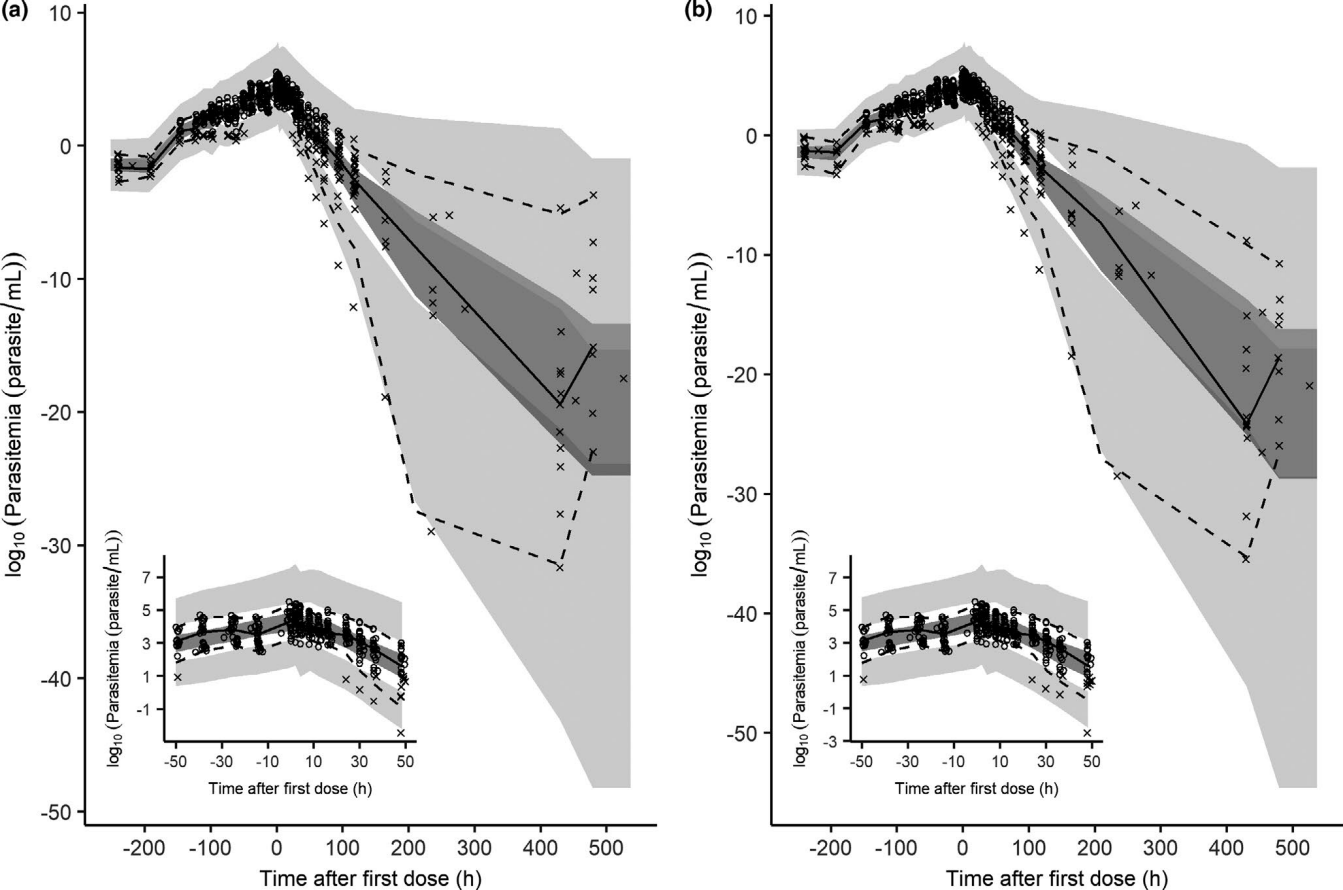
Visual predictive check of the final pharmacodynamic model of *Plasmodium vivax* parasitemia for chloroquine (**a**) plasma and (**b**) whole blood data. The insets show parasitemia simulations at −48 to 48 hours. The circles represent the observed data, the crosses represent the simulated values below the lower limit of quantification concentrations, the solid lines represent the 50th percentile of the observed data, the dashed lines represent the 50th and 95th percentiles for the observed data, the dark grey shaded areas represent the 95% confidence intervals for 50th percentile, and the light grey shaded area the 95% confidence interval for the 5th and 95th percentiles derived from 1,000 stochastic profiles simulated from the final population pharmacokinetic/pharmacodynamic model.

The goodness‐of‐fit plots did not show any systematic deviation between observed parasitemia and population and individual predicted parasitemias (**Figure** **S7**). The distributions of PWRES, IWRES, and NPDE plotted against population prediction and time after first chloroquine dose were homogenous around the zero line, with exception of a few observations, mainly data below the LOD that lay outside the −2 to 2 range. The major contributor of these observations was subject 205, who received his first dose of chloroquine on day 9. The estimated $E_{\text{max CQ}}$ for this subject is higher than the rest of subjects (0.365 hour^−1^ vs. others: 0.172–0.292 hour^−1^). The model predictive performance was confirmed with VPC plots (**Figure** **3**) in which the 5th, 50th, and 95th percentiles were within the 95% CI for both plasma and whole blood data.

### Simulations of chloroquine resistance

**Figure 4:**
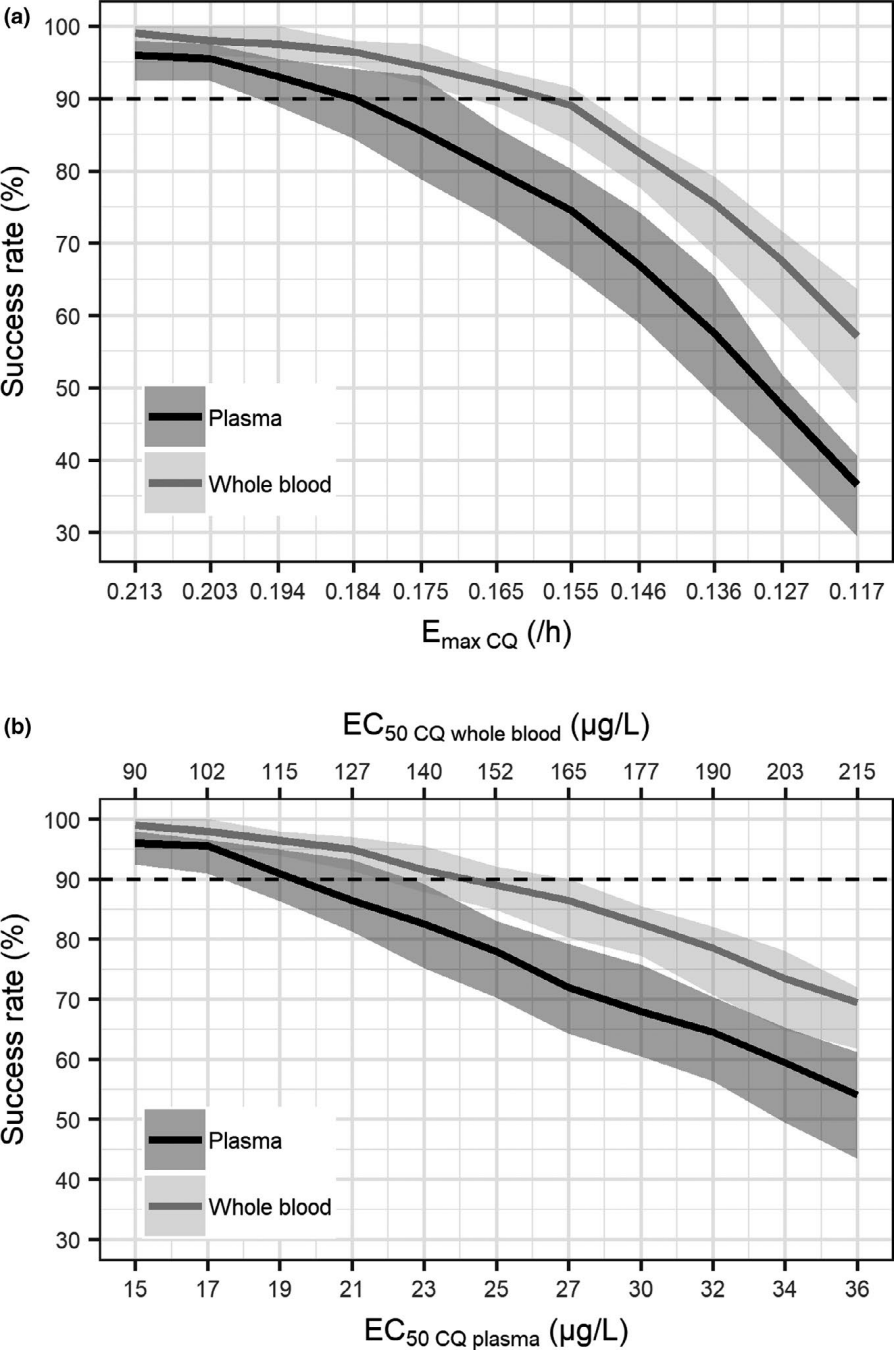
Simulated treatment success rate with (**a**) decreasing maximum parasite killing rate of chloroquine ( $E_{\max\text{CQ}}$) and (**b**) increasing chloroquine concentration at 50% of maximum killing rate ( ${\text{EC}}_{50\text{CQ biological matrix}}$). The black solid lines represent the median of simulated treatment success for chloroquine plasma data, the grey solid line represent the median of simulated treatment success for chloroquine whole blood data, the light grey shaded areas represent the simulated 95% confidence interval for the median of chloroquine plasma data, and the dark grey shaded areas represent the simulated 95% confidence interval for the median of chloroquine whole blood data.

Two scenarios of chloroquine resistance were considered. In the first scenario, simulations were performed by varying the $E_{\text{max CQ}}$ while holding constant the ${\text{EC}}_{50\;\text{CQ biological matrix}}$. **Figure** **4a** demonstrates that the treatment success rate at day 28 decreased as $E_{\text{max CQ}}$ decreased. The treatment success rate fell below 90% when $E_{\text{max CQ}}$ fell below 0.16 hour^−1^ for chloroquine plasma data, and to 0.184 hour^−1^ for chloroquine whole blood data. In the second scenario, the changes in parasite sensitivity to chloroquine was investigated by increasing the ${\text{EC}}_{50\;\text{CQ biological matrix}}$ while holding constant the $E_{\text{max CQ}}$ (**Figure** **4b**). As expected, the treatment success rate at day 28 decreased as the ${\text{EC}}_{50\;\text{CQ biological matrix}}$ increased. To achieve a treatment success rate above 90%, an ${\text{EC}}_{50\;\text{CQ}}$ of ≤ 19.5 µg/L (0.061 µmol/L) was required when using chloroquine plasma data, and of ≤ 146 µg/L (0.456 µmol/L) when using chloroquine whole blood data.

## DISCUSSION

This is the first study characterizing the PK/PD relationship of chloroquine plasma and whole blood concentrations on clearance of *P. vivax* parasites using a semimechanistic population PK/PD modeling. The final population PK model for plasma and whole blood samples was a two‐compartment model for both chloroquine and desethylchloroquine, with first‐order absorption and elimination. A delayed effect model was used to describe the effect of chloroquine on the *P. vivax* kinetics with equilibration half‐life of at least 24 hour. Given chloroquine’s long half‐life, this delayed effect is not clinically relevant in uncomplicated malaria. However, a delayed effect is undesirable for property of drugs used for treating severe malaria or for antimalarials with short half‐lives.^24^

The structural PK model described in this study was similar to two published studies.^9^, ^13^ However, another published study indicated a preference for one transit compartment absorption model over the first‐order absorption.^12^ Differences in sampling time points during the absorption phase may contribute to this discrepancy. These three published PK models were based on chloroquine and desethylchloroquine plasma concentrations.^9^, ^12^, ^13^ The PK parameter estimates of plasma samples in this study are slightly different in terms of volume of distribution and clearance with those previously reported.^9^, ^12^, ^13^ The apparent central volume of distribution ( $V_{\text{c CQ}}F)$ values reported in the published studies varied from 6.7–60.3 L/kg for chloroquine, and from 0.03–1.3 L/kg for desethylchloroquine (assuming a 70 kg individual). These values were comparable to the values estimated in this study ( $V_{\text{c CQ}}F$ = 41.9 L/kg; 95% CI 36.5–47.2; L/kg; $V_{\text{c DCQ}}F$ = 0.6 L/kg; 95% CI 0.1–1.0 L/kg). The apparent clearance of chloroquine ${\text{(CL}}_{\text{CQ}}F)$ estimated in this study was slightly higher at 0.78 L/h/kg (95% CI 0.71–0.85 L/h/kg) L/h/kg than the published values (range 0.09–0.5 L/h/kg; assuming a 70 kg individual). The apparent clearance of desethylchloroquine ( ${\text{CL}}_{\text{DCQ}}F$) value from literature ranged between 0.03 and 0.65 L/h/kg, which is within the range of the ${\text{CL}}_{\text{DCQ}}F$ value estimated in this study (0.54 L/h/kg; 95% CI 0.47–0.60 L/h/kg; assuming a 70 kg individual). This discrepancy may be attributable to different study populations, extensive PK sampling time points, differences in parasitemia levels, and use of concomitant antimalarial therapy. Published data on the population PK of hydroxychloroquine (a treatment currently being evaluated in clinical trials for the treatment of patients with coronavirus disease 2019 (COVID‐19)) are limited, with two studies reporting a one‐compartment PK model, probably due to limited sampling^25^, ^26^ and another study reporting a two‐compartment model.^27^ Compared with our estimates of the PK parameters, hydroxychloroquine has a smaller total apparent volume of distribution but was similar in regard to absorption and elimination parameters.

The analysis presented in this report represents the first attempt to develop a PK model of chloroquine and desethylchloroquine using whole blood concentrations. PK parameter estimates of whole blood samples were generally lower than that of plasma samples. This is due to differences in the partitioning of chloroquine and desethylchloroquine between erythrocytes and plasma. In this study, chloroquine and desethylchloroquine whole blood concentrations were higher than in plasma, which is in accordance with the results of other studies.^28^, ^29^, ^30^, ^31^ Consequently, the volume of distribution and clearance of chloroquine were sixfolds lower in whole blood than in plasma. Given the differences in PK parameter estimates between whole blood and plasma, the choice of biological matrix is important for monitoring chloroquine concentration. The World Health Organization recommendations are that whole blood be the preferred biological matrix for measurement of chloroquine levels, as it is more convenient to collect and process than packed red cells, plasma, or serum.^32^ Furthermore, factors such as the interval from sampling to centrifugation, duration, and force of centrifugation, do not influence chloroquine concentrations in whole blood.^30^, ^33^

The delay between appearance of chloroquine in plasma and whole blood and parasite killing was described using an effect compartment model. The utility of this delayed response model has been reported in modeling of *P. falciparum* malaria in patients receiving oral artesunate therapy.^34^ The PK/PD model developed in this study assumed that chloroquine is active against all asexual lifecycle stages of parasites. However, there is emerging evidence that *P. vivax* trophozoites and schizonts are insensitive to chloroquine.^35^, ^36^, ^37^ No recrudescence was observed for any subject, rendering the estimation of chloroquine ${\text{EC}}_{50}$ difficult. Chloroquine ${\text{EC}}_{50}$ for whole blood was fixed to a value previously reported in the literature based on the assumption that the drug sensitivity of parasites at the time of recrudescence is similar to that calculated from relapse data.^14^ ${\text{EC}}_{50\;\text{CQ plasma}}$ was assumed to be six folds lower than the ${\text{EC}}_{50\;\text{CQ whole blood}}$ based on whole blood to plasma ratio of chloroquine concentrations. Despite these limitations, this is the first study to characterize PK/PD relationship of chloroquine plasma and whole blood concentrations on clearance of *P. vivax* parasites. This is also the first time that the parasite killing effect of chloroquine was investigated in a *P. vivax* induced blood stage VIS.

The time above ${\text{EC}}_{50\;\text{CQ biological matrix}}$ was used as a PK determinant of chloroquine therapeutic outcome. With a log_10_ PRR_48_ of 2.6 (95% CI 2.5–2.7), 10 days are required for chloroquine to clear parasites in a hypothetical patient with a baseline parasitemia of 10.^12^ As chloroquine concentrations remain above ${\text{EC}}_{50}$ for 14–18 days, this ensures that all asexual parasites are removed from circulation, thereby curing the blood‐stage infection. The estimated log_10_ PRR_48_ in this study is similar to the reported values of chloroquine‐sensitive isolates (range 2.6–3.6),^38^, ^39^ mefloquine (2.6),^38^ and halofantrine (2.3).^38^ However, the log_10_ PRR_48_ estimated for chloroquine is lower than artesunate (3.2),^38^ artemether (3.2),^38^ artefenomel (range 4.6–6.3),^40^ and ganaplacide (range 3.1–3.8),^41^ but higher than primaquine (1.0),^38^ quinine (2.0),^38^ DSM265 (range 0.8–1.5),^42^ and sulfadoxine‐pyrimethamine (range 1.2–1.5)^38^ in patients with *P. vivax* malaria. Simulations showed that chloroquine efficacy was sensitive to changes in $E_{\max\text{CQ}}$_._ An $E_{\max\text{CQ}}$ reduction of 14% for plasma samples and 25% for whole blood samples resulted in treatment success rate of < 90%. In other reported *in silico* simulations, changes in $E_{\max\text{CQ}}$ were also found to have the largest effect on treatment outcome.^43^ Increasing the chloroquine dose in adults could restore the treatment success rate in areas with emerging chloroquine resistance but with an increased risk of development of toxicity. Of note, simulations of treatment success in this study were based on recrudescence within 28 days of follow‐up, and relapse within the treatment interval by reactivation of dormant hypnozoites was not taken into consideration.

In conclusion, we have successfully developed a population PK/PD model describing the PK and PD properties of chloroquine treatment in a VIS using the *P. vivax* induced blood‐stage malaria model. This model improves our understanding of the concentration‐effect relationship of chloroquine in *P. vivax* malaria and can be applied to optimize chloroquine dosing regimens. The PK/PD model of chloroquine developed, which includes estimation of parasite growth from samples collected pretreatment, can be used within a simulation framework to explore a range of scenarios. These include varying dosing schemes for chloroquine resistant *P. vivax* infection by varying the $E_{\max}$ or ${\text{EC}}_{50}$, the effect of incomplete adherence to the standard 3‐day dosing regimen on outcome, or varying the dosage schedule so as to minimize drug toxicity, which is primarily related to peak plasma concentration ( $C_{\max}$) could be simulated. Another model applicability, particularly the PK model, is the prediction of chloroquine dosing regimens for treatment of COVID‐19. Simulations of various dosing schemes using the developed PK model to achieve exposure above *in vitro* inhibition ${\text{EC}}_{50}$ of 1.13–7.36 µM^44^, ^45^, ^46^ at multiplicities of infection of 0.01–0.8 (assuming these values correspond to total plasma values) and putative whole blood *in vivo* ${\text{EC}}_{50}$ of 6.78–44.16 µM (based on whole blood‐to‐plasma ratio of 6:1 in this study), as well as prediction of chloroquine concentrations in the lungs (assuming similar lung‐to‐plasma correlations in animal studies^47^, ^48^) could be performed.

## FUNDING

This study was funded by Medicines for Malaria Venture (MMV). MMV is funded by a number of donors including USAID, Bill and Melinda Gates Foundation, UK Department for International Development, Norwegian Agency for Development Cooperation, Irish Aid, Newcrest Mining Limited, Australian Aid, Swiss Agency For Development and Co‐Operation, and Wellcome Trust. These funders had no role in the design, conduct, or analysis of the trial. J.S.M. was supported by an Australian National Health and Medical Research Foundation Practitioner Fellowship. J.A.S. is funded by an Australian National Health and Medical Research Council Senior Research Fellowship 1104975. This work was supported in part by the Australian Centre for Research Excellence in Malaria Elimination, funded by the NHMRC (1134989).

## Conflict of Interest

N.G., S.C., and J.J.M. are employees of Medicines for Malaria Venture (MMV); all other authors declared no competing interests for this work.

## Author Contributions

A.N.A.R., J.A.S., and J.S.M. wrote the manuscript. S.C., J.J.M., and J.S.M. designed the research. J.S.M. performed the research. A.N.A.R., L.M., N.G., and A.K. analyzed the data.

## Supporting information

Fig S1

Fig S2

Fig S3

Fig S4

Fig S5

Fig S6

Fig S7

Supplementary Material

## References

[1] World Health Organization . World Malaria Report 2019 <https://www.who.int/malaria/publications/world‐malaria‐report‐2019/en/>. (2019).

[2] Rieckmann, K.H. , Davis, D.R. & Hutton, D.C. Plasmodium vivax resistance to chloroquine? Lancet 2, 1183–1184 (1989). 2572903 10.1016/s0140-6736(89)91792-3

[3] Whitby, M. , Wood, G. , Veenendaal, J.R. & Rieckmann, K. Chloroquine‐resistant Plasmodium vivax. Lancet 334, 1395 (1989). 10.1016/s0140-6736(89)92002-3 2574333

[4] Price, R.N. , von Seidlein, L. , Valecha, N. , Nosten, F. , Baird, J.K. & White, N.J. Global extent of chloroquine‐resistant Plasmodium vivax: a systematic review and meta‐analysis. Lancet Infect. Dis. 14, 982–991 (2014). 25213732 10.1016/S1473-3099(14)70855-2 PMC4178238

[5] Commons, R.J. *et al*. The Vivax Surveyor: online mapping database for Plasmodium vivax clinical trials. Int. J. Parasitol. Drugs Drug Resist. 7, 181–190 (2017). 28384505 10.1016/j.ijpddr.2017.03.003 PMC5382033

[6] Edwards, G. , Looareesuwan, S. , Davies, A.J. , Wattanagoon, Y. , Phillips, R.E. & Warrell, D.A. Pharmacokinetics of chloroquine in Thais: plasma and red‐cell concentrations following an intravenous infusion to healthy subjects and patients with Plasmodium vivax malaria. Br. J. Clin. Pharmacol. 25, 477–485 (1988). 3289601 10.1111/j.1365-2125.1988.tb03332.x PMC1387810

[7] Na‐Bangchang, K. , Limpaibul, L. , Thanavibul, A. , Tan‐Ariya, P. & Karbwang, J. The pharmacokinetics of chloroquine in healthy Thai subjects and patients with Plasmodium vivax malaria. Br. J. Clin. Pharmacol. 38, 278–281 (1994). 7826832 10.1111/j.1365-2125.1994.tb04354.x PMC1364802

[8] Lee, S.J. *et al*. Chloroquine pharmacokinetics in pregnant and nonpregnant women with vivax malaria. Eur. J. Clin. Pharmacol. 64, 987–992 (2008). 18594802 10.1007/s00228-008-0500-z

[9] Karunajeewa, H.A. *et al*. Pharmacokinetics of chloroquine and monodesethylchloroquine in pregnancy. Antimicrob. Agents Chemother. 54, 1186–1192 (2010). 20086162 10.1128/AAC.01269-09 PMC2825967

[10] Kadam, P.P. , Gogtay, N.J. , Karande, S. , Shah, V. & Thatte, U.M. Evaluation of pharmacokinetics of single‐dose chloroquine in malnourished children with malaria‐ a comparative study with normally nourished children. Indian J. Pharmacol. 48, 498–502 (2016). 27721533 10.4103/0253-7613.190720 PMC5051241

[11] Pereira, D. *et al*. Safety, efficacy and pharmacokinetic evaluations of a new coated chloroquine tablet in a single‐arm open‐label non‐comparative trial in Brazil: a step towards a user‐friendly malaria vivax treatment. Malar J 15, 477 (2016). 27639847 10.1186/s12936-016-1530-0 PMC5027105

[12] Hoglund, R. , Moussavi, Y. , Ruengweerayut, R. , Cheomung, A. , Abelo, A. & Na‐Bangchang, K. Population pharmacokinetics of a three‐day chloroquine treatment in patients with Plasmodium vivax infection on the Thai‐Myanmar border. Malar. J. 15, 129–137 (2016). 26928448 10.1186/s12936-016-1181-1 PMC4772585

[13] Salman, S. *et al*. Optimal antimalarial dose regimens for chloroquine in pregnancy based on population pharmacokinetic modelling. Int. J. Antimicrob. Agents 50, 542–551 (2017). 28669839 10.1016/j.ijantimicag.2017.05.011

[14] Watson, J. , Chu, C.S. , Tarning, J. & White, N.J. Characterizing blood‐stage antimalarial drug MIC values in vivo using reinfection patterns. Antimicrob. Agents Chemother. 62, e02476–e2517 (2018). 29661873 10.1128/AAC.02476-17 PMC6021672

[15] Collins, K.A. *et al*. A Plasmodium vivax experimental human infection model for evaluating the efficacy of interventions. J. Clin. Invest.. doi:10.1172/JCI134923. [epub ahead of print]. PMC7259989 32045385

[16] McCarthy, J.S. *et al*. Experimentally induced blood‐stage Plasmodium vivax infection in healthy volunteers. J. Infect. Dis. 208, 1688–1694 (2013). 23908484 10.1093/infdis/jit394 PMC3888148

[17] R Core Team . R: A language and environment for statistical computing. (R Foundation for Statistical Computing, Vienna, Austria, 2018).

[18] Wickham, H. ggplot2: Elegant graphics for data analysis <https://ggplot2‐book.org/> (2016).

[19] Aderounmu, A.F. In vitro assessment of the antimalarial activity of chloroquine and its major metabolites. Ann. Trop. Med. Parasitol. 78, 581–585 (1984). 6398033 10.1080/00034983.1984.11811868

[20] Fu, S. , Bjorkman, A. , Wahlin, B. , Ofori‐Adjei, D. , Ericsson, O. & Sjoqvist, F. In vitro activity of chloroquine, the two enantiomers of chloroquine, desethylchloroquine and pyronaridine against Plasmodium falciparum. Br. J. Clin. Pharmacol. 22, 93–96 (1986). 3527245 PMC1401091

[21] Geary, T.G. , Divo, A.A. & Jensen, J.B. Activity of quinoline‐containing antimalarials against chloroquine‐sensitive and ‐resistant strains of Plasmodium falciparum in vitro. Trans. R. Soc. Trop. Med. Hyg. 81, 499–503 (1987). 3318024 10.1016/0035-9203(87)90175-1

[22] Verdier, F. , Le Bras, J. , Clavier, F. & Hatin, I. Blood levels and in vitro activity of desethylchloroquine against Plasmodium falciparum. Lancet 1, 1186–1187 (1984). 10.1016/s0140-6736(84)91436-3 6144914

[23] Beal, S.L. Ways to fit a PK model with some data below the quantification limit. J. Pharmacokinet. Pharmacodyn. 28, 481–504 (2001). 11768292 10.1023/a:1012299115260

[24] Khoury, D.S. , Cromer, D. , Mohrle, J.J. , McCarthy, J.S. & Davenport, M.P. Defining the effectiveness of antimalarial chemotherapy: investigation of the lag in parasite clearance following drug administration. J. Infect. Dis. 214, 753–761 (2016). 27252475 10.1093/infdis/jiw234 PMC4978373

[25] Carmichael, S.J. , Charles, B. & Tett, S.E. Population pharmacokinetics of hydroxychloroquine in patients with rheumatoid arthritis. Ther. Drug Monit. 25, 671–681 (2003). 14639053 10.1097/00007691-200312000-00005

[26] Morita, S. , Takahashi, T. , Yoshida, Y. & Yokota, N. Population pharmacokinetics of hydroxychloroquine in Japanese patients with cutaneous or systemic lupus erythematosus. Ther. Drug Monit. 38, 259–267 (2016). 26587870 10.1097/FTD.0000000000000261

[27] Lim, H.S. *et al*. Pharmacokinetics of hydroxychloroquine and its clinical implications in chemoprophylaxis against malaria caused by Plasmodium vivax. Antimicrob. Agents Chemother. 53, 1468–1475 (2009). 19188392 10.1128/AAC.00339-08 PMC2663072

[28] Frisk‐Holmberg, M. , Bergqvist, Y. , Termond, E. & Domeij‐Nyberg, B. The single dose kinetics of chloroquine and its major metabolite desethylchloroquine in healthy subjects. Eur. J. Clin. Pharmacol. 26, 521–530 (1984). 6610555 10.1007/BF00542151

[29] Nosal, R. , Ericsson, O. , Sjoqvist, F. & Durisova, M. Distribution of chloroquine in human blood fractions. Meth. Find Exp. Clin. Pharmacol. 10, 581–587 (1988). 3226224

[30] Bergqvist, Y. & Domeij‐Nyberg, B. Distribution of chloroquine and its metabolite desethyl‐chloroquine in human blood cells and its implication for the quantitative determination of these compounds in serum and plasma. J. Chromatogr. 272, 137–148 (1983). 6841533 10.1016/s0378-4347(00)86110-1

[31] Gustafsson, L.L. *et al*. Disposition of chloroquine in man after single intravenous and oral doses. Br. J. Clin. Pharmacol. 15, 471–479 (1983). 6849784 10.1111/j.1365-2125.1983.tb01532.x PMC1427807

[32] World Health Organization . Methods and techniques for assessing exposure to antimalarial drugs in clinical field studies <https://www.who.int/malaria/publications/atoz/9789241502061/en/> (2011).

[33] Rombo, L. , Ericsson, O. , Alvan, G. , Lindstrom, B. , Gustafsson, L.L. & Sjoqvist, F. Chloroquine and desethylchloroquine in plasma, serum, and whole blood: problems in assay and handling of samples. Ther. Drug Monit. 7, 211–215 (1985). 4024216 10.1097/00007691-198506000-00013

[34] Lohy Das, J.P. *et al*. Population pharmacokinetic and pharmacodynamic properties of artesunate in patients with artemisinin sensitive and resistant infections in Southern Myanmar. Malar. J. 17, 126 (2018). 29566683 10.1186/s12936-018-2278-5 PMC5865368

[35] Russell, B. *et al*. Determinants of in vitro drug susceptibility testing of Plasmodium vivax. Antimicrob. Agents Chemother. 52, 1040–1045 (2008). 18180357 10.1128/AAC.01334-07 PMC2258486

[36] Sharrock, W.W. *et al*. *Plasmodium vivax* trophozoites insensitive to chloroquine. Malar. J. 7, 94 (2008). 18505560 10.1186/1475-2875-7-94 PMC2430579

[37] Kerlin, D.H. *et al*. An analytical method for assessing stage‐specific drug activity in Plasmodium vivax malaria: implications for ex vivo drug susceptibility testing. PLoS Negl. Trop. Dis. 6, e1772 (2012). 22880143 10.1371/journal.pntd.0001772 PMC3413709

[38] Pukrittayakamee, S. *et al*. Therapeutic responses to different antimalarial drugs in vivax malaria. Antimicrob. Agents Chemother. 44, 1680–1685 (2000). 10817728 10.1128/aac.44.6.1680-1685.2000 PMC89932

[39] Phan, G.T. *et al*. Artemisinin or chloroquine for blood stage Plasmodium vivax malaria in Vietnam. Trop. Med. Int. Health 7, 858–864 (2002). 12358621 10.1046/j.1365-3156.2002.00948.x

[40] Phyo, A.P. *et al*. Antimalarial activity of artefenomel (OZ439), a novel synthetic antimalarial endoperoxide, in patients with Plasmodium falciparum and Plasmodium vivax malaria: an open‐label phase 2 trial. Lancet Infect. Dis. 16, 61–69 (2016). 26448141 10.1016/S1473-3099(15)00320-5 PMC4700386

[41] White, N.J. *et al*. Antimalarial activity of KAF156 in falciparum and vivax malaria. N. Engl. J. Med. 375, 1152–1160 (2016). 27653565 10.1056/NEJMoa1602250 PMC5142602

[42] Llanos‐Cuentas, A. *et al*. Antimalarial activity of single‐dose DSM265, a novel plasmodium dihydroorotate dehydrogenase inhibitor, in patients with uncomplicated Plasmodium falciparum or Plasmodium vivax malaria infection: a proof‐of‐concept, open‐label, phase 2a study. Lancet Infect. Dis. 18, 874–883 (2018). 29909069 10.1016/S1473-3099(18)30309-8 PMC6060173

[43] Winter, K. & Hastings, I.M. Development, evaluation, and application of an in silico model for antimalarial drug treatment and failure. Antimicrob. Agents Chemother. 55, 3380–3392 (2011). 21537019 10.1128/AAC.01712-10 PMC3122393

[44] Liu, J. *et al*. Hydroxychloroquine, a less toxic derivative of chloroquine, is effective in inhibiting SARS‐CoV‐2 infection in vitro. Cell Discov 6, 16 (2020). 32194981 10.1038/s41421-020-0156-0 PMC7078228

[45] Wang, M. *et al*. Remdesivir and chloroquine effectively inhibit the recently emerged novel coronavirus (2019‐nCoV) in vitro. Cell Res. 30, 269–271 (2020). 32020029 10.1038/s41422-020-0282-0 PMC7054408

[46] Yao, X. *et al*. In vitro antiviral activity and projection of optimized dosing design of hydroxychloroquine for the treatment of severe acute respiratory syndrome coronavirus 2 (SARS‐CoV‐2). Clin. Infect. Dis. 10.1093/cid/ciaa237 PMC7108130 32150618

[47] Adelusi, S.A. & Salako, L.A. Kinetics of the distribution and elimination of chloroquine in the rat. Gen. Pharmacol. 13, 433–437 (1982). 7173598 10.1016/0306-3623(82)90110-0

[48] Adelusi, S.A. & Salako, L.A. Tissue and blood concentrations of chloroquine following chronic administration in the rat. J. Pharm. Pharmacol. 34, 733–735 (1982). 6129306 10.1111/j.2042-7158.1982.tb06211.x

