# Supplementary material for: Population Pharmacokinetics and Pharmacodynamics of Chloroquine in a Plasmodium vivax Volunteer Infection Study
Source: Clin Pharmacol Ther. 2020 Jul 2;108(5):1055–66. doi: 10.1002/cpt.1893 (PMC7276750; doi:10.1002/cpt.1893)

Figure S1 Plots of chloroquine and desethylchloroquine concentrations in plasma, whole blood and erythrocytes over time, and correlations between chloroquine and desethylchloroquine whole blood and erythrocyte concentrations.

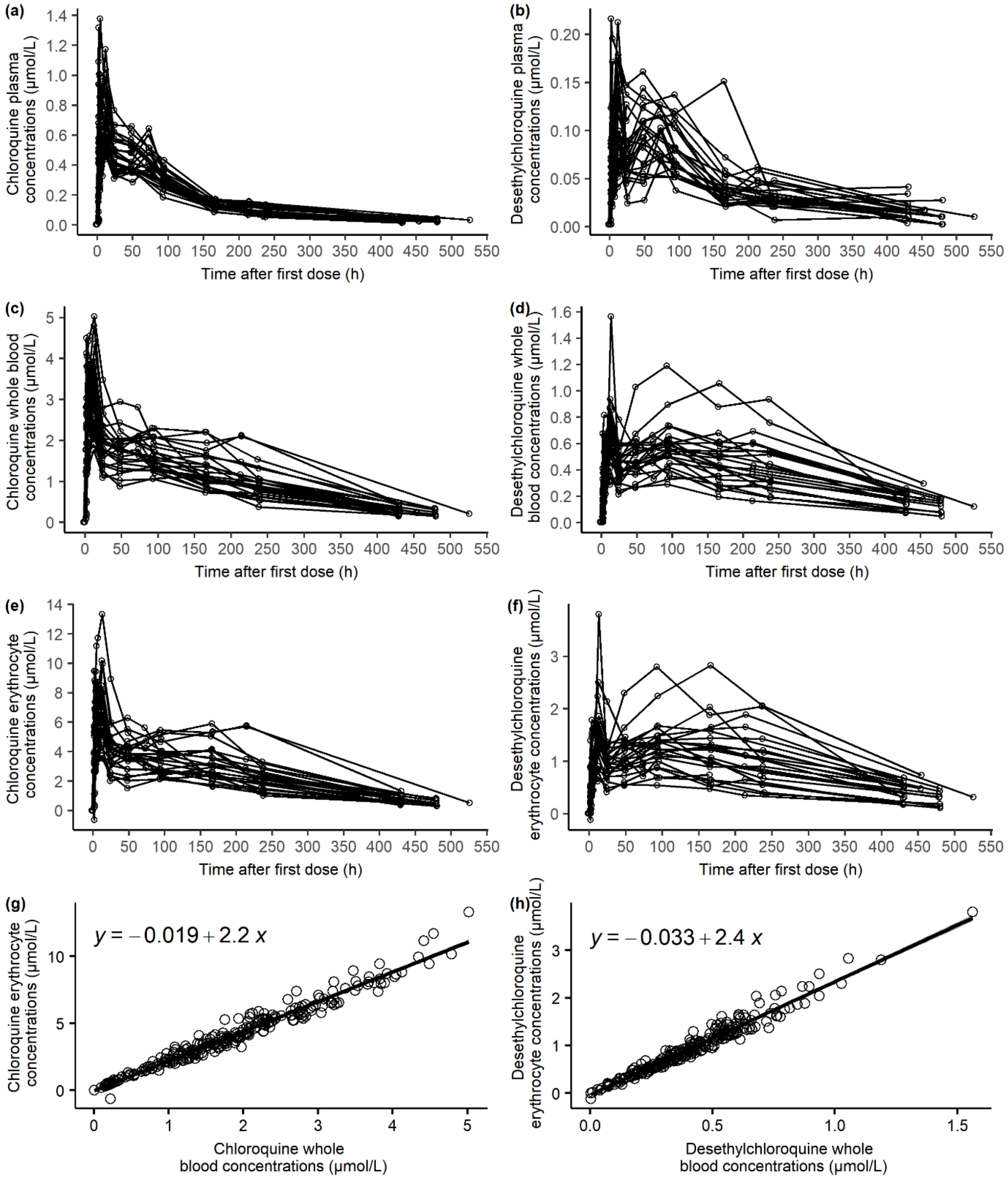

Supplement: Supplementary file 1 — Fig S1 [file CPT-108-1055-s007.pdf]
