# Supplementary material for: Population Pharmacokinetics and Pharmacodynamics of Chloroquine in a Plasmodium vivax Volunteer Infection Study
Source: Clin Pharmacol Ther. 2020 Jul 2;108(5):1055–66. doi: 10.1002/cpt.1893 (PMC7276750; doi:10.1002/cpt.1893)

**Figure S7** Goodness of fit plots of the final population pharmacodynamic model for *P. vivax* parasitemia for chloroquine whole blood data. The circles represent the data above the lower limit of quantification and the crosses represent the simulated values below the lower limit of quantification data. PWRES, population weighted residuals; IWRES, individual weighted residuals; NPDE, normalized prediction distribution errors.

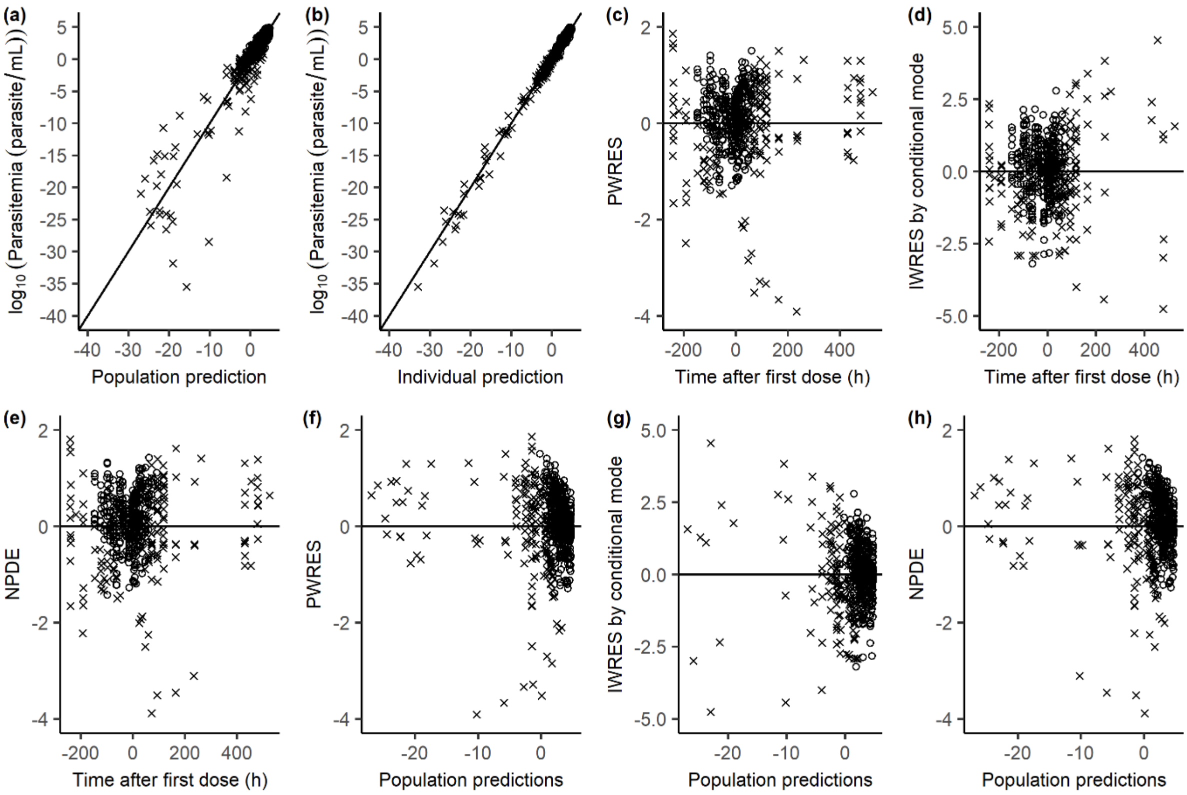

Supplement: Supplementary file 7 — Fig S7 [file CPT-108-1055-s005.pdf]
