# Supplementary material for: Population Pharmacokinetics and Pharmacodynamics of Chloroquine in a Plasmodium vivax Volunteer Infection Study
Source: Clin Pharmacol Ther. 2020 Jul 2;108(5):1055–66. doi: 10.1002/cpt.1893 (PMC7276750; doi:10.1002/cpt.1893)
Supplement: Supplementary file 8 — Supplementary Material [file CPT-108-1055-s004.docx]

**Population pharmacokinetics and pharmacodynamics of chloroquine in a *Plasmodium vivax* volunteer infection study.**

**Supplementary Supporting Information**

**METHODS**

**Study design and subjects**

*Plasmodium vivax* parasitemia was measured using quantitative real-time polymerase chain reaction (qPCR). For Cohort 1, samples of all time-points were stored for each subject and tested retrospectively in a single assay at completion of the cohort. For Cohorts 2 and 3, samples were tested during the trial in a single assay performed daily. Unpublished data showed that parasitemia values, parasitemia multiplication rate estimate and parasite clearance rate estimates were consistent when the qPCR assay was performed retrospectively and daily (Ballard E, Wang C, Marquart L, Gaydon J, Jennings H, Bialasiewicz S, et al).

**Data used for analysis**

Chloroquine and desethylchloroquine concentrations in erythrocytes were calculated based on the following equation:

$\boldsymbol{C}_{\boldsymbol{whole blood}}\boldsymbol{=}\left( \boldsymbol{1-H}\boldsymbol{CT} \right)\boldsymbol{\times}\boldsymbol{C}_{\boldsymbol{plasma}}\boldsymbol{+HCT \times}\boldsymbol{C}_{\boldsymbol{erythrocytes}}$

where C_whole blood_ was the drug concentrations in whole blood, C_plasma_ was the drug concentrations in plasma, C_erythrocyte_ was the drug concentrations in erythrocytes and HCT was the subject’s hematocrit. Hematocrit levels were measured throughout the study period. Correlations between drug concentrations in whole blood and erythrocytes were analysed using Spearman’s correlation.

**Population pharmacokinetic modelling**

Inter-individual variability (IIV) and inter-occasion variability of the PK parameters were assumed to be normally distributed on log-scale:

$$\theta_{i}= \theta\cdot e^{\eta_{i}}$$

where $\theta_{i}$ was the parameter for the i^th^ subject, $\theta$ was the typical value of the parameter in the population and $\eta_{i}$ was a subject-specific random effect with the mean of 0 and variance $\omega^{2}$. In the case where a random effect was found to be independently distributed and did not correlate with another random effect, the variance-covariance matrix (**Ω**) was expressed as a diagonal matrix in which the corresponding off-diagonal components of **Ω** were set to 0:

$$\boldsymbol{\Omega}= \left[ \begin{matrix} {\omega^{2}}_{\theta_{1}} & \\ 0 & {\omega^{2}}_{\theta_{2}} \end{matrix} \right]$$

where ${\omega^{2}}_{\theta_{1}}$ was the variance for parameter $\theta_{1}$and ${\omega^{2}}_{\theta_{2}}$ was the variance for parameter $\theta_{2}$. If the parameter $\theta_{1}$and parameter $\theta_{2}$ were correlated random effects, then the **Ω** matrix was written, in the simplest case of two random effects, as:

$$\boldsymbol{\Omega}= \left[ \begin{matrix} {\omega^{2}}_{\theta_{1}} & \\ {\omega^{2}}_{\theta_{2},\theta_{1}} & {\omega^{2}}_{\theta_{2}} \end{matrix} \right]$$

where ${\omega^{2}}_{\theta_{2},\theta_{1}}$was the covariance between parameter $\theta_{1}$and parameter $\theta_{2}$. The correlation (defined as $\rho)$between parameter $\theta_{1}$and parameter $\theta_{2}$ was calculated as:

$$\rho= \frac{{\omega^{2}}_{\theta_{2,}\theta_{1}}}{\sqrt{{\omega^{2}}_{\theta_{2}}\times{\omega^{2}}_{\theta_{1}}}}$$

The residual unexplained variability (RUV) was tested using an additive, proportional or combined error model:

| Additive : $y_{\mathrm{ij}}= ŷ_{\mathrm{ij}}+\hat{\epsilon}_{\mathrm{ij}}$ |
| --- |
| Proportional : $y_{\mathrm{ij}}= ŷ_{\mathrm{ij}}\times(1+\hat{\epsilon}_{\mathrm{ij}})$ |
| Combined : $y_{\mathrm{ij}}= ŷ_{\mathrm{ij}}\times\left( 1+\hat{\epsilon}_{ij1} \right)+\hat{\epsilon}_{ij2}$ |

where $y_{ij}$ and $ŷ_{ij}$ represented the observed and predicted concentration, respectively, for the *i*^th^ subject at the *j*^th^ timepoint, $\hat{\epsilon}_{ij}$ was the residual variability which was normally distributed with mean 0 and variance σ^2^. For combined error model, $\hat{\epsilon}_{ij1}$ and $\hat{\epsilon}_{ij2}$ corresponded to proportional and additive error models, respectively.

The relationships between PK parameters and potential continuous or categorical covariates were evaluated using:

Continuous covariate:

$$\theta_{pop,cov}= \theta_{\mathrm{pop}}\left( \frac{\mathrm{COV}}{\mathrm{COV}_{\mathrm{median}}} \right)^{\beta}$$

Categorical covariate:

$$\theta_{pop,cov}= \theta_{pop,ref} e^{\beta}$$

where $\theta_{pop,cov}$ was the population estimates with covariate effect, $\theta_{\mathrm{pop}}$ was the typical population estimate, $\theta_{pop,ref}$ was the population estimate for population of reference of the categorical covariate, COV was the continuous covariate, COV_median_ was the median of the continuous covariate and β was the covariate effect. With regards to allometric scaling, the exponents of 0.75 and 1 were used for clearance and volume, respectively, in order to relate PK parameters with body weight.

**Population pharmacokinetic-pharmacodynamic modelling**

In this analysis, the changes in parasitemia over time in the presence of chloroquine are assumed to be a result of the difference between parasite growth rate ($k_{\mathrm{grow}}$) and the rate at which parasites are killed by chloroquine ($k_{\mathrm{kill}}$) with a baseline parasitemia ($P_{\mathrm{base}}$) at time t_0_ (time of first observation). The equations were expressed in log-scale such as:

$$\left\{ \begin{matrix} \frac{\mathrm{dPL}}{\mathrm{dt}} & = & k_{\mathrm{grow}}-k_{\mathrm{kill}} \\ & & \\ \mathrm{PL}\left( t_{0} \right) & = & PL_{\mathrm{base}} \end{matrix} \right.$$

where PL was the log_10_-transformed parasite counts.

Different structural PK-PD models were tested to assess the effect of chloroquine on the parasite killing. The $E_{\max}$ model assumed a direct effect of chloroquine plasma and whole blood concentrations on parasite killing rate:

$$k_{\mathrm{kill}}=\frac{E_{\max}\times{C_{\mathrm{drug}}}^{\mathrm{Hill}}}{{\mathrm{EC}_{50}}^{\mathrm{Hill}}+ {C_{\mathrm{drug}}}^{\mathrm{Hill}}}$$

where $E_{\max}$ represented the maximum parasite killing attributable to the drug, $\mathrm{EC}_{50}$ represented the drug concentration producing 50% of the $E_{\max}$, Hill represented the Hill coefficient which described the steepness of the concentration-effect curve and $C_{\mathrm{drug}}$ was the concentration of the drug.

The turnover model assumed an indirect effect of chloroquine plasma and whole blood concentrations on parasite killing rate due to lag in the PD processes (i.e., the kinetics of drug-receptor binding):

$$\left\{ \begin{matrix} k_{\mathrm{kill}} & = & E_{\max}\times R \\ \frac{\mathrm{dR}}{\mathrm{dt}} & = & k_{\mathrm{in}}\cdot\left( \frac{{C_{\mathrm{drug}}}^{\mathrm{Hill}}}{{\mathrm{EC}_{50}}^{\mathrm{Hill}}+ {C_{\mathrm{drug}}}^{\mathrm{Hill}}}-R \right) \end{matrix} \right.\phantom{\left. \right\}}$$

where $k_{\mathrm{in}}$ represented the turnover rate for a delayed effect.

The effect compartment model assumed an indirect effect of chloroquine plasma and whole blood concentration on parasite killing rate due to biophase equilibration. This model can be interpreted as the delay for the drug to reach site of action in which the chloroquine concentrations in the effect compartment drives the drug effect:

$$\left\{ \begin{matrix} k_{\mathrm{kill}} & = & \frac{E_{\max}\times{C_{e}}^{\mathrm{Hill}}}{{\mathrm{EC}_{50}}^{\mathrm{Hill}}+{C_{e}}^{\mathrm{Hill}}} \\ \frac{dC_{e}}{\mathrm{dt}} & = & k_{e}\cdot\left( C_{c}-C_{e} \right) \end{matrix} \right.$$

where $k_{e}$ represented the transfer rate constant between the central and effect compartments and $C_{e}$ was the drug concentration in the effect compartment.

Individual PD parameters were obtained using empirical Bayes estimates. The parasites were assumed to grow exponentially, expressed as parasite multiplication rate per asexual cycle of 48 h ($\mathrm{PMR}_{48}$) as given by:

$$\mathrm{PMR}_{48}= e^{(k_{\mathrm{grow}}\times48h)}$$

The parasite reduction ratio over 48 h ($\mathrm{PRR}_{48}$) was determined by calculating the difference between the simulated log parasitemia number at start of treatment (0 h) and the number remaining 48 h later. The parasite clearance half-life ($\mathrm{PCt}_{½}$, h) was calculated as:

$$\mathrm{PCt}_{½}= \frac{Ln(2)}{E_{\max}- k_{\mathrm{grow}}}$$

Time above $\mathrm{EC}_{50 CQ biological matrix}$ was estimated by determining the duration of time simulated chloroquine plasma or whole blood concentrations exceeded the $\mathrm{EC}_{50}$.

**Simulations of chloroquine resistance**

A 100-fold decrease in $\mathrm{PRR}_{48}$ was selected as lower limit (worst case scenario) of $E_{\max\mathrm{CQ}}$ in which the relationship between $E_{\max\mathrm{CQ}}$ and log_10_ $\mathrm{PRR}_{48}$ (assuming chloroquine plasma and whole blood concentrations were well above minimum parasiticidal concentration for this time span) was described as:

$$\log_{10} \mathrm{PRR}_{48}= \frac{(E_{\max\mathrm{CQ}}- k_{\mathrm{grow}})}{Ln(10)}\times48$$

where $k_{\mathrm{grow}}$ was the parasite growth rate constant. Eleven equally spaced values between 0.117 and 0.213 h^-1^ were selected, where the upper limit represents the population mean estimate derived from the PKPD modelling.

For $\mathrm{EC}_{50 CQ whole blood}$ an upper limit of 215 µg/L (≈ 0.672 µmol/L) was chosen which equates to approximately 20% of the maximum chloroquine whole blood concentration ($C_{\max}$: 1075 µg/L ≈ 3.36 µmol/L) of the simulated population PK profiles. Eleven equally spaced values between 90 and 215 µg/L were selected for $\mathrm{EC}_{50 CQ whole blood}$. The $\mathrm{EC}_{50 CQ plasma}$ values were selected based on assumption that $\mathrm{EC}_{50 CQ plasma}$ was six folds lower than $\mathrm{EC}_{50 CQ whole blood}$. Accordingly, the upper limit of $\mathrm{EC}_{50 CQ plasma}$ was approximately 14% of the $C_{\max}$ (256 µg/L ≈ 0.8 µmol/L) of the simulated population PK profiles.

The baseline parasitemia at the time of initial treatment was assumed to have a log-normal distribution with a median value of 10^7.1^ parasites/mL and a standard deviation of 0.28, characteristic of values observed in a phase IIa study undertaken in Thailand.^1^ For each chloroquine resistance scenario, plasma and whole blood concentration-time and parasitemia-time profiles up to Day 28 for 1,000 hypothetical patients were simulated. In these chloroquine resistant simulations, population parameters were sampled from the uncertainty distribution and individual parameters were sampled from IIV. Dosing regimen recommended by World Health Organization (WHO) was used for the simulations (10 mg base/kg initially, followed by 10 mg base/kg on the second day and 5 mg base/kg on the third day for 60 kg patients).^2^ Treatment success was defined as absence of parasitemia on Day 28. A threshold value of 90% was selected for percentage of patients with treatment success as recommended by WHO.^2^

**RESULTS**

**Population pharmacokinetic modelling**

The effect of body weight on apparent clearance of chloroquine ($\mathrm{CL}_{\mathrm{CQ}}/F$) and desethylchloroquine ($\mathrm{CL}_{\mathrm{DCQ}}/F$) was not significant due to the narrow range of body weight in our study population. Chloroquine relative bioavailability ($F_{\mathrm{rel}}$) in plasma decreased by 18% in male than in female subjects and increased by 15% when body weight decreased from 71 kg to 56 kg (average body weight for adults in six countries which contributed to 85% of estimated *P. vivax* malaria in 2018).^3, 4^ For whole blood samples, male subjects had 12% $\mathrm{CL}_{\mathrm{CQ}}/F$ and 5% higher apparent central volume of distribution of chloroquine ($V_{c CQ}/F$) than female subjects and $F_{\mathrm{rel}}$ increased by 18% when body weight decreased from 71 kg to 56 kg. Clinical relevance assessment revealed that the 95% CI of these covariate effects did not lie between ±20% predetermined region. This indicates that the covariates were unlikely to be clinically important, and thus were not included in the final PK model.

(1) Phyo, A.P. *et al.* Antimalarial activity of artefenomel (OZ439), a novel synthetic antimalarial endoperoxide, in patients with *Plasmodium falciparum* and *Plasmodium vivax* malaria: an open-label phase 2 trial. *Lancet Infect Dis* **16**, 61-9 (2016).

(2) World Health Organization. *Guidelines for treatment of malaria. Third edition* (2015).

(3) Walpole, S.C., Prieto-Merino, D., Edwards, P., Cleland, J., Stevens, G. & Roberts, I. The weight of nations: an estimation of adult human biomass. *BMC Public Health* **12**, 10.1186/1471-2458-12-439 (2012).

(4) World Health Organization. *World Malaria Report 2019* (2019).
